# A peculiar foreign body ingestion in 2-year-old girl complicated by esophageal perforation: case report and review of the literature

**DOI:** 10.1093/omcr/omae040

**Published:** 2024-05-20

**Authors:** Danah Albarrak, Suliman Alrajhi, Mohammed Naeem

**Affiliations:** College of Medicine, King Saud bin Abdulaziz University for Health Sciences, Riyadh, Saudi Arabia; Department of Radiology, King Abdulaziz Medical City, Ministry of National Guard Health Affairs, Riyadh, Saudi Arabia; Pediatric Intensive Care Department, King Abdullah Specialized Children’s Hospital, King Abdulaziz Medical City, Ministry of National Guard Health Affairs, Riyadh, Saudi Arabia

## Abstract

Foreign body ingestion is a common pediatric gastrointestinal emergency, which should be suspected in all patients who present with signs of airway obstruction or upper GI bleeding, especially if it developed after the child was left unwitnessed for a while. The most common foreign bodies identified in the literature are button batteries or coins. Early identification and management of suspected foreign body ingestion is crucial as it can lead to devastating complications including bleeding, fistula formation, perforation, mediastinitis, or abscess. Here we report a case of a peculiar foreign body ingestion resulting in esophageal perforation in a 2-year-old girl.

## INTRODUCTION

Foreign body (FB) ingestion is considered a common gastrointestinal (GI) emergency in the pediatric population with the majority of cases occurring in children aged 6 months to 5 years [1, 2]. The most common FB identified are coins, however, button batteries, magnets, toys, jewelry, and less commonly sharp objects have been reported in the literature [1, 2]. Based on the 2021 Annual American Association of Poison Control Center report, FB ingestion in children less than 5 years accounted for approximately 55 000 cases with higher rates observed among boys [3]. Clinical presentation varies depending on the type and location of the FB ingested however common symptoms may include drooling, vomiting, dysphagia, throat, or chest pain [4]. Fortunately, the mortality rate is low and the majority of FBs ingested are spontaneously passed [1]. However, based on current guidelines endoscopic retrieval is indicated if the FB is impacted in the esophagus within 2hrs in case of battery ingestion with or without symptoms. A coin, magnet, or sharp foreign bodies impacted in the esophagus in an asymptomatic patient can be removed within 24hrs [5]. Any long foreign (more than 6-cm) in the esophagus should be removed within 24hrs even if the patient is asymptomatic. Only < 1% of patients may require further surgical intervention [1]. Possible complications of FB ingestion include the development of ulceration, bleeding, fistula formation, perforation, mediastinitis, or abscess [1]. Here we report a case of esophageal perforation caused by FB ingestion in a 2-year-old girl.

## CASE PRESENTATION

A 2-year-old developmentally normal girl with no significant past medical or surgical history presented to the emergency department (ED) with a three-day history of vomiting large amounts of fresh blood with clots and a single episode of dark stool. The family denied any history of fever, abdominal pain, upper respiratory tract symptoms, bleeding from other sites, easy bruising, or trauma. Two days prior to this presentation the family sought medical attention in another hospital, the patient was found to have a low hemoglobin (Hb) level of 5 g/dl. She was suspected to have foreign body ingestion and was recommended to proceed with an endoscopy. However, the family refused and the patient was discharged against medical advice.

**Figure 1:**
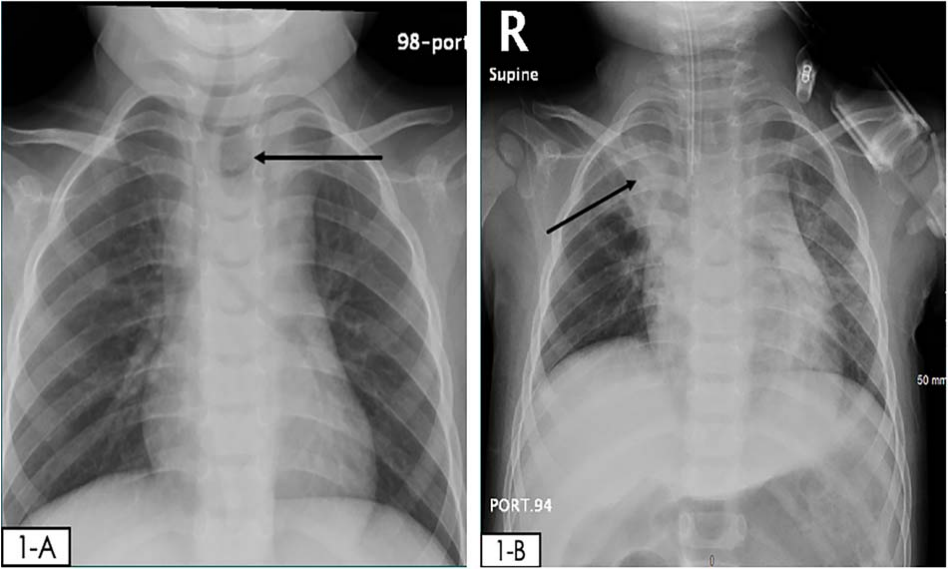
(**A**) initial CXR in the ED: reveals a round radiolucent object seen lateral to the trachea on the left side with mild widening of the mediastinum and clear lung fields. (**B**) Repeated CXR 4hrs after, an endotracheal tube is observed and there is an opacity in the right upper lung likely due to aspiration.

**Figure 2:**
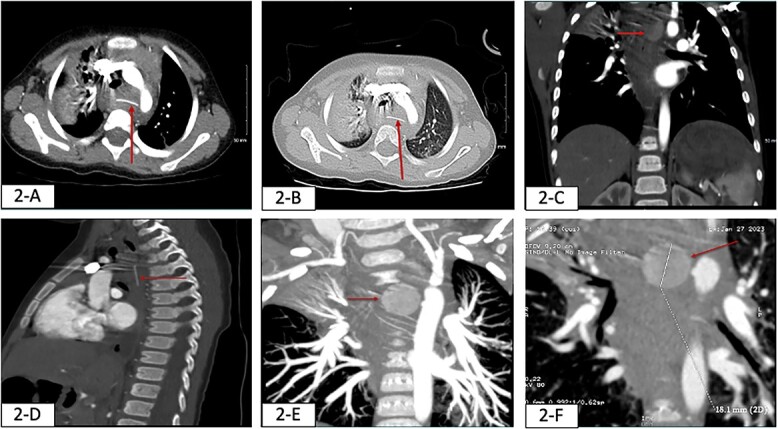
Findings of chest CT on the day of patient presentation (red arrows). (**A**) axial view: shows hyperdense object seen in the posterior mediastinum likely within the esophagus. (**B**) Axial and lung window view: hyperdense subject seen in the posterior mediastinum and there is consolidation in the right upper lobe (aspiration). (**C**) Coronal view: hyperdense object (FB) seen in the posterior mediastinum. (**D**) Sagittal view: hyperdense FB seen in the posterior mediastinum. Maximum intensity projection (MIP) imaging revealed a round hyperdense FB measure it 18.1 mm seen in the posterior mediastinum in multiple views, (**E**) Coronal view, (**F**) sagittal view.

Upon physical examination, vital signs were as follows HR 127 beats/minute, BP 84/39 mmHg, RR 26 breaths/minute, afebrile, and oxygen saturation 98% on room air. The patient was alert and active, with intact pulses, and warm extremities with no bleeding, bruises, or skin changes; the rest of the physical examination was unremarkable. The repeated Hb level was 4 g/dl, thus the patient received 10 ml/kg (-O) packed red blood cells (PRBC) transfusion. Initial chest x-ray (CXR) revealed a round radiolucent object seen lateral to the trachea on the left side with mild widening of the mediastinum, and lung fields were clear (Fig. 1). The patient was anticipated to have a difficult airway and therefore was intubated to proceed with imaging safely, CXR was repeated revealing an opacity in the right upper lung likely due to aspiration (Fig. 1). Computed tomography (CT) showed a hyperdense object measuring 18.1 mm, seen in the posterior mediastinum, consolidation in the right upper lobe due to aspiration and mediastinal hematoma (Fig. 2).

The patient was rushed for exploratory thoracotomy and FB retrieval with concurrent esophagogastroduodenoscopy (EGD). Initially, rigid endoscopy was performed and revealed pooling of blood inside the esophageal lumen with bulging of the mucosa, there was no active bleeding. Left thoracotomy revealed multiple perforated feeding vessels to the pleura, which were ligated using a clip. There was no evidence of fistula, active bleeding, or inflammatory process in that area. The esophagus was skeletonized and showed no pus collection, hematoma, or cyst. However, it couldn’t be skeletonized more proximally as the arch of the aorta was intervening. The area was reexamined using rigid endoscopy and there was no evidence of perforation or active bleeding: therefore; proceeded with a fluoroscopic esophagogram which showed an esophageal pouch at the left second rib, suspicious of esophageal duplication cyst. The gastrology department was consulted to perform flexible endoscopy, which revealed a cystic-like lesion in the esophagus at almost 15 cm from the mouth with no evidence of perforation, guidewire was inserted in the cyst for the surgeon to continue.

**Figure 3:**
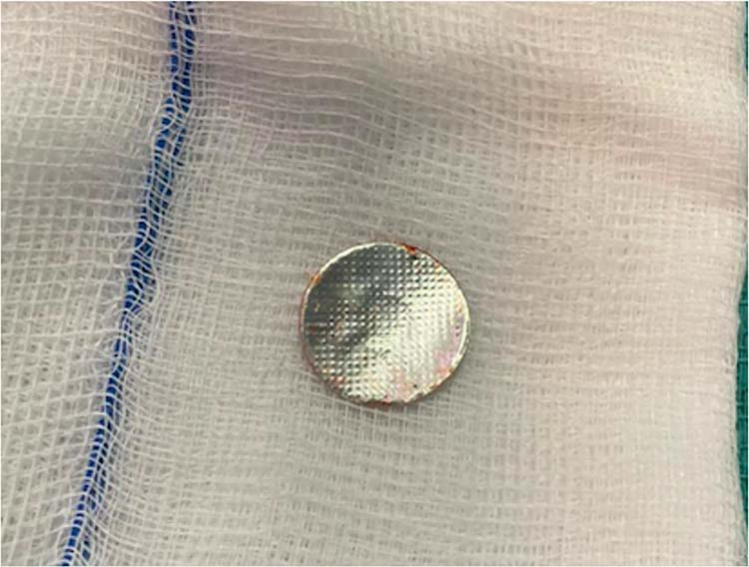
Picture of the foreign body after retrieval.

Left thoracotomy was closed and converted to right thoracotomy which revealed a cystic-like structure, esophageal pouch, which was flimsy and macerated. An enterotomy was performed in the anterior esophageal wall almost 3 cm in length. The esophageal lumen was examined from the inside, and old digested blood coming from the esophageal lumen was noted: however; there was no active bleeding. Additionally, the cyst was followed to its origin and proximal to the enterotomy made, upon closer inspection and dissection the FB was found eroding the esophagus coming from the esophageal mucosa which was easily retrieved (Fig. 3). The operation was concluded by a primary repair of the esophageal side perforation and repair of the esophageal enterotomy.

**Figure 4:**
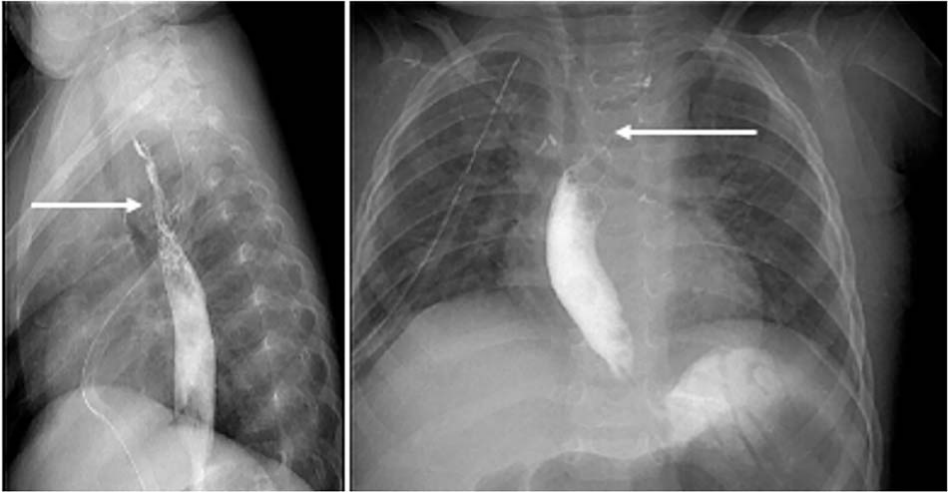
Fluoroscopy on day 7 post-op: revealed a narrowing of the upper esophagus (white arrows) with passing contrast to the lower esophagus and stomach.

Following the surgery, the patient was admitted to PICU for close monitoring. Initially, the patient was doing well: however; on the third day she suddenly developed emesis and epistaxis and had systolic hypotension, tachycardia, and tachypnea. She improved after being managed with 20 ml/kg stat bolus, PRPCs, and platelet transfusion. Additionally, she developed left followed by right-sided pneumothorax which improved the next day. Moreover, the patient’s blood culture came back positive for gram cocci in chains (anginosus group), infectious disease department was consulted and recommended treatment with tazocin. Fluoroscopy was performed seven days postop revealing a narrowing of the upper esophagus with passing contrast to the lower esophagus and stomach (Fig. 4). On day 12, the patient improved clinically and was discharged.

## DISCUSSION

FB ingestion is frequently encountered in pediatric emergencies, patients might present with a wide array of symptoms including drooling, dysphagia, vomiting, or emesis [4]. Timely identification of FB and management is essential to avoid possible complications which can include but are not limited to esophageal ulceration, bleeding, fistula formation, and perforation [1].

Based on the review of the literature 12 cases reported esophageal perforation secondary to FB ingestion in children aged 24 months or less (Table 1). The most common FBs were button batteries and metal objects, none of the FBs identified was similar to the one retrieved from our patient. Delayed presentation and prolonged duration of impaction, especially > 1 week resulted in the development of multiple complications. Most patients were alive and well on follow-up with no complications, 2 lost follow-up, and 2 developed complications including vocal cord paralysis and intermittent croup [6–11].

**Table 1:** Literature review of esophageal perforation secondary to FB ingestion in children aged 24 months or less

Year	Author	Type of FB	Age	Gender	Duration of impaction	Complications	Follow up
2016	Kristina Leinwand, et al.	Button battery	24-months	Boy	6 h	Focal perforation of the upper esophagus	Lost follow-up
			11-months	Boy	27 h	Vocal cord paralysis, localized esophageal perforation	vocal cord paralysis
2020	Priya V. Shah, et al.	Metal ornament hook	10-months	Girl	2 months	Esophageal perforation, diffuse mediastinitis, brain abscess, seizure	Alive and well, all complications treated and resolved.
2006	Ming-Yu Chang, et al.	Fish bone	7-month	Boy	1 week	Pneumomediastinum, persistent retropharyngeal air collection, pneumothorax, multilocular empyema, mediastinitis, and esophageal perforation, Horner’s syndrome.	Alive and well, all complications treated and resolved.
2010	Stanley J. Kimball, et al.	Button battery	9-months	Girl	4 weeks	Small contained posterior esophageal perforation, esophageal stricture with single dilatation at 3 1/2 months	Alive and well, all complications treated and resolved.
			13-months	Boy	1 week	Complete perforation of the anterior esophagus and the posterior trachea, TEF formation	Intermittent croup 3½ years after the injury, reflux esophagitis
2013	Nicholas J. Panella, et al.	Button battery	8-months	Boy	1 week	Esophageal perforation with neck abscess	Lost follow up, but lastly seen asymptomatic.
2015	Nitin James Peters, et al.	Metal screw	24-months	Not specified	Unknown	Esophageal perforation	Alive and well, all complications treated and resolved.
		Metal hairpin	5-months		4 days		
		Metal coin	11-months		Unknown		
		Glass marble	19-months		4-5 months		
		Metal coin	21-months		Unknown		

In our case, the patient had delayed presentation of FB ingestion as she started to develop signs of Upper GI bleeding 3 days prior. CT confirmed the presence of a round hyperdense FB in the posterior mediastinum. Flexible endoscopy was only able to identify a spot in the esophagus that looked cystic-like lesion. An emergency thoracotomy retrieved the FB that had eroded the esophagus. This foreign body was found to be a cork that is widely used in the caps of disposable bottles. Postoperatively the patient was admitted to the PICU for close monitoring and later discharged once she was clinically stable.

FB ingestion should be suspected in all pediatric patients who present with signs of airway obstruction or upper GI bleeding, especially if it developed after the child was left unwitnessed for a while. Parental education regarding prevention, signs and symptoms of FB ingestion is imminent in order to prevent delayed presentation and the development of devastating complications.

## CONCLUSION

Primary care and emergency healthcare workers should consider FB ingestion as a differential diagnosis in any pediatric patient presenting with symptoms of airway compromise or GI bleeding, especially if there is a history of leaving the child unchaperoned. Additionally, community education on the topic is recommended to allow early recognition, and treatment and to avoid the development of any complications.
